# The clinical and epidemiological characteristics of a series of patients living with HIV admitted for COVID-19 in a district hospital

**DOI:** 10.1186/s12879-023-08004-6

**Published:** 2023-02-28

**Authors:** Ayanda Trevor Mnguni, Denzil Schietekat, Nabilah Ebrahim, Nawhaal Sonday, Nicholas Boliter, Neshaad Schrueder, Shiraaz Gabriels, Lovemore N. Sigwadhi, Annalise E. Zemlin, Zivanai C. Chapanduka, Veranyuy Ngah, Anteneh Yalew, Thumeka Jalavu, Ibtisam Abdullah, Jacques L. Tamuzi, Yamanya Tembo, Mary-Ann Davies, Rene English, Peter S. Nyasulu

**Affiliations:** 1 Department of Medicine, Faculty of Medicine and Health Sciences, Stellenbosch University, Cape Town, South Africa; 2 Khayelitsha District Hospital, Cape Town, South Africa; 3 Division of Epidemiology and Biostatistics, Department of Global Health, Faculty of Medicine and Health Sciences, Stellenbosch University, Cape Town, South Africa; 4 Division of Chemical Pathology, Department of Pathology, Faculty of Medicine and Health Sciences, Stellenbosch University and NHLS Tygerberg Hospital, Cape Town, South Africa; 5 Division of Haematological Pathology, Department of Pathology, Faculty of Medicine and Health Sciences, Stellenbosch University and NHLS Tygerberg Hospital, Cape Town, South Africa; 6 Division of Haematological Pathology, Department of Pathology, Northland District Health Board, Northland, New Zealand; 7 Health Impact Assessment Directorate, Western Cape Government, Cape Town, South Africa; 8 Centre for Infectious Disease Epidemiology and Research, School of Public Health and Family Medicine, University of Cape Town, Cape Town, South Africa; 9 School of Public Health and Family Medicine, University of Cape Town, Cape Town, South Africa; 10 Division of Health Systems and Public Health, Department of Global Health, Faculty of Medicine and Health Sciences, Stellenbosch University, Cape Town, South Africa; 11 Division of Epidemiology and Biostatistics, Faculty of Medicine and Health Sciences, School of Public Health, University of the Witwatersrand, Johannesburg, South Africa

**Keywords:** COVID-19, SARS-CoV-2, HIV, PLWH, TB, District hospital

## Abstract

**Background:**

The coronavirus disease 2019 (COVID-19) pandemic continues to evolve. Globally, COVID-19 continues to strain even the most resilient healthcare systems, with Omicron being the latest variant. We made a thorough search for literature describing the effects of the COVID-19 in a high human immunodeficiency virus (HIV)/tuberculosis (TB) burden district-level hospital setting. We found scanty literature.

**Methods:**

A retrospective observational study was conducted at Khayelitsha District Hospital in Cape Town, South Africa (SA) over the period March 2020–December 2021. We included confirmed COVID-19 cases with HIV infection aged from 18 years and above. Analysis was performed to identify predictors of mortality or hospital discharge among people living with HIV (PLWH). Predictors investigated include CD4 count, antiretroviral therapy (ART), TB, non-communicable diseases, haematological, and biochemical parameters.

**Findings:**

This cohort of PLWH with SARS-CoV-2 infection had a median (IQR) age of 46 (37–54) years, male sex distribution of 29.1%, and a median (IQR) CD4 count of 267 (141–457) cells/mm3. Of 255 patients, 195 (76%) patients were discharged, 60 (24%) patients died. One hundred and sixty-nine patients (88%) were on ART with 73(28%) patients having acquired immunodeficiency syndrome (AIDS). After multivariable analysis, smoking (risk ratio [RR]: 2.86 (1.75–4.69)), neutrophilia [RR]: 1.024 (1.01–1.03), and glycated haemoglobin A1 (HbA1c) [RR]: 1.01 (1.007–1.01) were associated with mortality.

**Conclusion:**

The district hospital had a high COVID-19 mortality rate among PLWH. Easy-to-access biomarkers such as CRP, neutrophilia, and HbA1c may play a significant role in informing clinical management to prevent high mortality due to COVID-19 in PLWH at the district-level hospitals.

## Background

Globally, COVID-19 continues to test even the resilient healthcare systems, with the Omicron being the latest variant in circulation. As of March 25th, 2022, the World Health Organization (WHO) had reported over 476 million confirmed cases of COVID-19, including over 6 million deaths, and approximately 11 billion vaccine doses had been administered [1]. South Africa, a middle-income country, is experiencing epidemics of noncommunicable diseases and chronic infectious diseases such as the human immunodeficiency virus (HIV) and tuberculosis (TB). Around eight million of South Africa's 60 million people are HIV-positive, accounting for one-fifth of all HIV-positive people worldwide [2, 3]. A high proportion of people newly diagnosed in South Africa are at an advanced stage of HIV infection (defined as a CD4 count < 200 cells/mm^3^), by which point the immune system is extremely compromised [3, 4]. Lastly, South Africa recorded 0.7% of TB prevalence for all ages, among them 301,000 are new cases of TB [5].

A systematic review including twenty-two studies conducted in developed and low and middle countries showed that HIV remains a significant risk factor for SARS-CoV-2 infection and is linked to a higher risk of death from COVID-19 [6]. Similarly, a meta-analysis found that active TB was more common in COVID-19/HIV co-infected people than in COVID-19 infected people [7]. On the other hand, COVID-19 risk was high among current HIV/TB co-infected cases [7]. According to one study, patients with SARS-CoV-2 infection who also had HIV and TB had altered T-cell functions and were at higher risk of developing severe disease [8]. Furthermore, COVID-19 could be accelerated in HIV patients with compromised immunity [7, 9]. SARS-CoV-2 and HIV may both decrease lymphocyte and CD4 counts. Corticosteroids given as management for moderate to severe disease for SARS-CoV-2 may predispose to TB [7, 10]. Co-infection with SARS-CoV-2 and tuberculosis may exacerbate the pathologies associated with each pathogen [11]. Co-infection of macrophages can increase the production of pro- and anti-inflammatory cytokines, playing a significant role in pathogenesis of COVID-19 [11]. COVID-19 pneumonia, on the other hand, may hasten the progression of tuberculosis [12, 13]. The combination of a weakened virus-induced cytokine response caused by a severe/dysfunctional immune system and in vitro activity of certain antiretroviral drugs (tenofovir and lopinavir-ritonavir) on coronaviruses is thought to be associated with a reduction in the severity COVID-19 in PLWH [14–16]. However, PLWH were 30% more likely to die after admission to hospital with COVID-19, regardless of age, gender, severity at presentation, or co-morbidities [17]. Diabetes, high blood pressure, and male sex were all associated with an increased risk of death [18]. These comorbidities are caused by ART-related inflammation and ongoing immune dysregulation, which may influence COVID-19 disease severity, the durability of protective antiviral responses, and vaccine responsiveness [19–22]. According to a recent non-peer reviewed systematic review, COVID-19 vaccines had lower immunogenicity and antigenicity among PLWH, than among HIV negative people [23]. Furthermore, PLWH require more attention during the COVID-19 pandemic because the emergence of Omicron in Southern Africa has raised the question of whether this heavily mutated variant is the result of the HIV pandemic, a common cause of immunodeficiency in the region [3].

South Africa has a dual healthcare system with both public and privately funded facilities. The publicly funded district healthcare system serves approximately 84% of the population [24]. The district-level healthcare system, which is often understaffed, along with the primary healthcare system, are the primary points of contact for COVID-19 patients. Experiences from district-level hospitals with high HIV/TB burdens provide a unique opportunity to study the effects of the COVID-19 pandemic at the 'grass roots' level, which will inform the success or failure of public health interventions. The purpose of this study was to describe the clinical features of COVID-19 patients in a district hospital setting in a population with a high TB/HIV prevalence.

## Methods

### Study design

This was a retrospective observational study describing the epidemiological and clinical characteristics of COVID-19 patients admitted at Khayelitsha District Hospital, Cape Town, South Africa from March 2020–December 2021.

### Case definition and management

A COVID-19 case was defined as PLWH with a positive antigen or reverse transcriptase polymerase chain reaction (RT-PCR) assay (Abbott Panbio Covid-19 antigen test or Cepheid Xpert Xpress CoV-2 plus test) for SARS-CoV-2 who were admitted to hospital. For those diagnosed with HIV, ELISA (BIOMERIEUX MINI VIDAS duo kit) test was performed on all patient during admissions if HIV status was not known. In terms of management and treatment, all patients were given oxygen and corticosteroid therapy. The management pillars were based on the recovery trial results, which showed that COVID-19 patients who were oxygen dependent and on corticosteroid therapy had a better outcome than those who were not on corticosteroid therapy. Because the clinical and radiological findings were consistent with hypoxemic pneumonia, all patients were started on empiric bacterial treatment with Ceftriaxone and Azithromycin in accordance with national and international guidelines for community acquired pneumonia. Once COVID-19 was confirmed by antigen testing or RT-PCR, empiric antibiotic therapy was thereafter immediately discontinued.

### Study population

We included all consecutive COVID-19 cases with HIV infection, 18 years and older who required hospital admission from March 2020 until December 2021. The main indication for hospitalization was anyone with COVID-19 pneumonia requiring oxygen therapy. Patients were prospectively followed up until completed hospital course (either discharge, transfer to tertiary or field hospital or death) at censoring. A total of 255 PLWH were included in this study.

### Setting

**Fig. 1:**
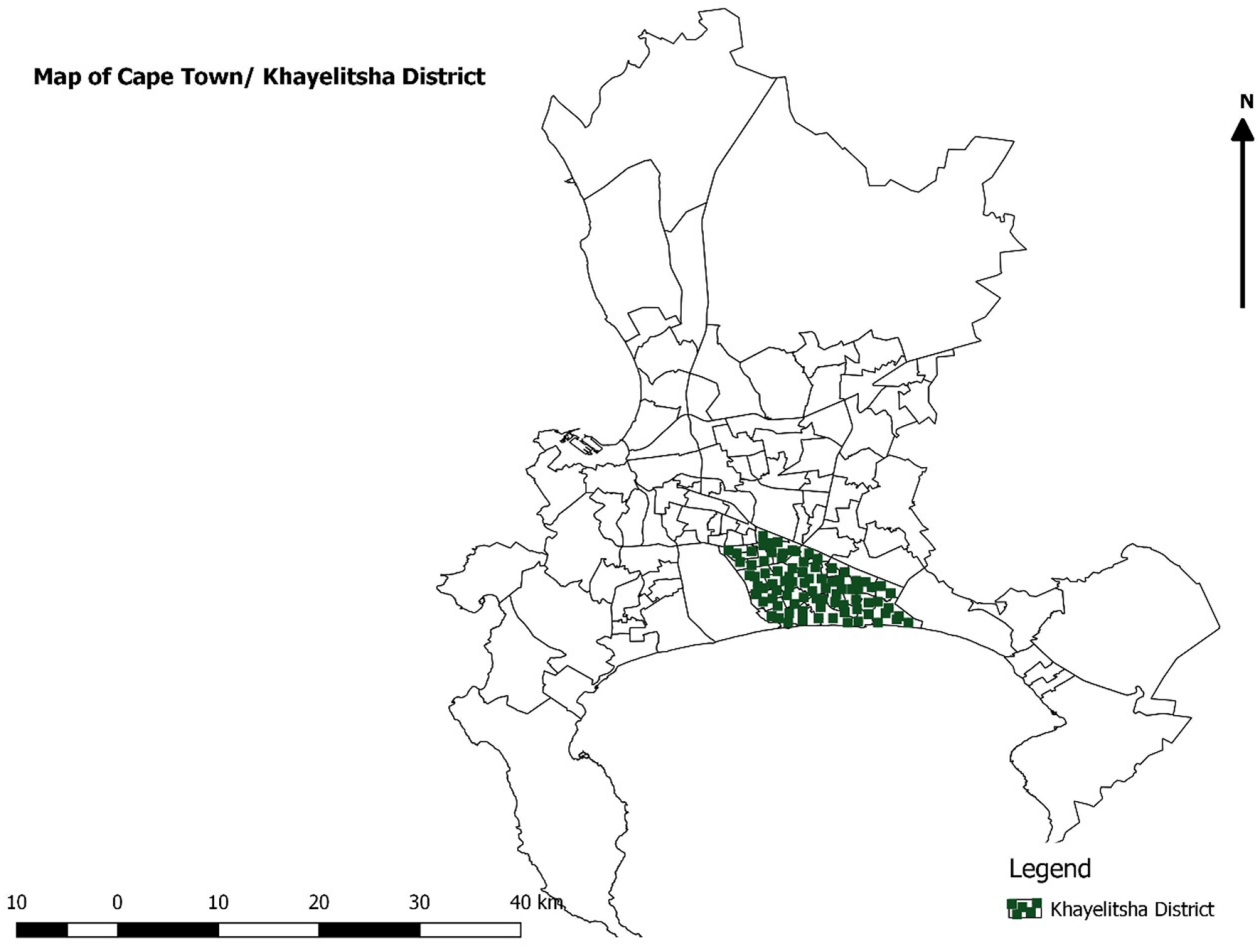
Map of the district of Khayelitsha in Cape Town

Khayelitsha District Hospital, located in Mandela Park, Khayelitsha, is a 330-bed district hospital that opened in 2012. Khayelitsha township is Southeast of Cape Town (Fig. 1). Most of the population (98.6%) is black African [25]. Khayelitsha was created by the apartheid government via the forced removals of the Group Areas Act. It is isolated, located on sand dunes, and has a substantial risk of flooding. It is entirely residential, with no designated commercial or industrial areas [26]. Most of the population (55.6%) lives in informal housing [27]. Khayelitsha has South Africa's highest concentration of poverty and unemployment [27]. Furthermore, with 37.0% TB burden and 47.6% HIV/TB co-infection, Khayelitsha has the worst health indicators in the Cape Town metropolitan area [28]. Similarly, Khayelitsha has the highest rates of mortality for stroke, hypertension, and diabetes mellitus [29, 30]. Overpopulation, poverty, trauma, mental health, communicable, and noncommunicable diseases have all posed significant challenges to Khayelitsha Hospital's ability to serve the community. No fewer than ten nearby primary care clinics refer patients to the hospital. The population of Khayelitsha is estimated to be close to 500 000 people [31], but this is a gross underestimation due to the community's continued growth. In this population, 51.1% are females, 48.8% are between the ages of 25 and 65, only 4.9% completed high school, and 30.7% finished secondary school [27]. The unemployment rate is estimated to be 38.32% [27]. The first case of COVID-19 in a South African township occurred in Khayelitsha. Overtime medical admissions at Khayelitsha District Hospital have risen steadily over the last five years, averaging 5500 per year with a mortality rate of less than 10%.

### Data collection

The patients’ demographic, clinical, baseline laboratory results and outcome data were collected from digital application-based registries. Additional data were captured from electronic medical records. Furthermore, the presence of comorbidities including HIV/AIDS hypertension, diabetes, overweight or obesity (defined as a body mass index (BMI > 25 or > 30 kg/m^2^ respectively) or as documented by treating clinicians as the BMI was not captured. Other comorbidities were cardiac disease, chronic kidney disease, and active or previous history of TB were also included in the data collection process. Baseline arterial blood gas and laboratory values including severity indices were captured. These included the partial pressure of arterial oxygen to fraction of inspired oxygen (P/F Ratio), the white cell count (WCC), the neutrophil to lymphocyte ratio (N/L Ratio), serum creatinine (Cr), HbA1c and the CRP and CD_4_ cell counts. Viral load measurements were captured if they were performed up to a year prior to admission. The main outcome of the study was death or discharge. Patients whose outcome data was unknown were excluded from the analyses. Data were captured retrospectively based on clinical notes at the bedside, which were securely stored electronically, and clinical data were entered remotely onto a Research Electronic Data Capture (REDCap®) database; laboratory results were imported into the database. Data were checked by the ‘data entry supervisor.’

### Ethical approval

Ethical approval for the study was obtained from the Stellenbosch University health research ethics committee (Ethics Reference Number: N20/05/020_COVID-19).

### Statistical analysis

Continuous variables were expressed as median with inter-quartile range for skewed data. Categorical variables were expressed using frequencies and percentages. A multivariable model was developed for demographics, comorbidities, clinical symptoms, haematological, and biochemical parameters using variables strongly associated with mortality or survival outcomes at univariable analysis. The comparison between mortality and survival used the Pearson *χ*^2^ test or Fisher exact test where appropriate for categorical variables, and the Wilcoxon's rank-sum test for continuous variables. Robust Poisson regression was used to assess significant associations between demographic, laboratory results, and mortality. Factors associated with death at p < 0.15 in unadjusted univariable robust Poisson regression were included in a multivariable model to identify predictor variables associated with death. Due to the high mortality, around 24%, the logistic regression overestimated the effect measure with large standard errors resulting in wide confidence intervals, therefore robust Poisson regression was used. Adjusted risk ratios and their 95% CIs were used as a measure of association. Receiver Operating Characteristic curve (ROC) analysis was performed to evaluate the diagnostic performance of various haematological and biochemical parameters to discriminate between severe cases in terms of survival and non-survival. Schoenfeld residuals and cox proportional hazards test were used to assess the proportional hazards assumption [32]. Kaplan-Meir survival curve was plotted, and the log-rank test was used to compare the two groups [32]. All statistical analyses were performed using Stata (V.16, Stata Corp, College Station, Texas, USA) and R (V, 4.1.0, R Core Team) with R Studio (V.1.3, R Studio Team) statistical software.

## Results

### Characteristics of PLWH with SARS-COV-2

A total of 255 PLWH were admitted during the first and the second waves. The cohort was comprised mainly of females (70.9%) and mostly above 50 years old (38.8%), with 55.2% (n = 32) of all patients who died being 50 years and older (Table 1). Among PLWH, the main pre-existing co-morbidities were hypertension (43.1%), diabetes mellitus (29.4%), chronic kidney disease (7.1%), TB (12.9%), and acute kidney injury (16.9%) (Table 1). The presenting clinical signs and symptoms suggestive of COVID-19 included fever (38.8%), cough (75.7%), sore throat (11.0%), and myalgia (22.4%) (Table 1). Most of this PLWH cohort (88.5%) were on ARV therapy. Among them, 36.9% of this cohort had severe immunosuppression with CD4 < 200mm3 with only 21% having a CD4 ≥ 500 mm^3^ (Table 1). The median oxygen saturation and PaO2 were 93% (86–97) and 8 kPa (6.8–9.9), respectively (Table 1). Higher median CRP was observed among those who died 20.4 (12.4–28.5) as compared to those discharged 13.9 (7.0–20.7) characterised this PLWH cohort and, high median (IQR) HbA1c was a predictor of mortality with 12% (10.35–13.35%) (Table 1). The case fatality rate in the study population was 23.5% (60/255). Around sixty-seven percent (150/225) had known HIV viral load and seventy-eight (117/150) percent had undetectable HIV viral load (< 50 copies/ml).

**Table 1:** Comparison of COVID-19 characteristics among PLWH who died or survived

Variable	Level	Total (N = 255), n (%)	Discharge (N = 195), n (%)	Death (N = 60), n (%)	p
^Age	Median	46 (37–54)	45 (36–52)	51 (44–59)	< 0.001
Age categories	< 40	77 (30.2)	68 (34.9)	9 (15.0)	0.003
	40–50	83 (32.6)	64 (32.8)	19 (31.7)	
	> 50	95 (37.2)	63 (32.3)	32 (53.3)	
Sex	Male	74 (29.1)	50 (25.8)	24 (40.0)	0.034
	Female	180 (70.9)	144 (74.2)	36 (60.0)	
Smoker	No	242 (94.9)	190 (97.4)	52 (86.7)	0.003
	Yes	13 (5.1)	5 (2.6)	8 (13.3)	
Hypertension	No	145 (56.9)	115 (59.0)	30 (50.0)	0.220
	Yes	110 (43.1)	80 (41.0)	30 (50.0)	
Diabetes Mellitus	No	180 (70.6)	141 (72.3)	39 (65.0)	0.280
	Yes	75 (29.4)	54 (27.7)	21 (35.0)	
Chronic Kidney Disease	No	237 (92.9)	183 (93.8)	54 (90.0)	0.390
	Yes	18 (7.1)	12 (6.2)	6 (10.0)	
TB	No	222 (87.1)	172 (88.2)	50 (83.3)	0.330
	Yes	33 (12.9)	23 (11.8)	10 (16.7)	
Congestive Heart Failure	No	248 (97.3)	191 (97.9)	57 (95.0)	0.360
	Yes	7 (2.7)	4 (2.1)	3 (5.0)	
Acute kidney injury	No	212 (83.1)	170 (87.2)	42 (70.0)	0.002
	Yes	43 (16.9)	25 (12.8)	18 (30.0)	
ARDS	No	244 (95.7)	187 (95.9)	57 (95.0)	0.720
	Yes	11 (4.3)	8 (4.1)	3 (5.0)	
Shock	No	248 (97.3)	190 (97.4)	58 (96.7)	0.670
	Yes	7 (2.7)	5 (2.6)	2 (3.3)	
Cough	No	62 (24.3)	46 (23.6)	16 (26.7)	0.630
	Yes	193 (75.7)	149 (76.4)	44 (73.3)	
Fever	No	153 (61.2)	115 (60.2)	38 (64.4)	0.560
	Yes	97 (38.8)	76 (39.8)	21 (35.6)	
Sore Throat	No	226 (89.0)	171 (87.7)	55 (93.2)	0.230
	Yes	28 (11.0)	24 (12.3)	4 (6.8)	
Myalgia	No	198 (77.6)	150 (76.9)	48 (80.0)	0.620
	Yes	57 (22.4)	45 (23.1)	12 (20.0)	
ARV therapy	No	22 (11.5)	17 (11.6)	5 (11.1)	1.000
	Yes	169 (88.5)	129 (88.4)	40 (88.9)	
Viral load	Undetectable (< 50 copies)	117 (78.0)	87 (79.1)	23 (20.9)	0.593
	Undetectable (< 50 copies)	33 (22.0)	30 (75.0)	10 (25.0)	
	Unknown/missing	105	85	20	
*CD4 categories	< 200	73 (36.9)	48 (32.0)	25 (52.1)	0.021
	200–499	83 (41.9)	65 (43.3)	18 (37.5)	
	$\ge$500	42 (21.2)	37 (24.7)	5 (10.4)	
*CD4 Count	Median (IQR)	267 (141–457)	296.5 (174–498)	188 (72.5–337)	0.004
Oxygen saturation	Median (IQR)	93 (86–97)	94 (90–98)	86.5 (74.5–93)	< 0.001
PaO2	Median (IQR)	8 (6.8–9.9)	8.1 (6.9–9.9)	7.3 (6.1–11.2)	0.160
FiO2	Median (IQR)	21 (21–21)	21 (21–21)	21 (21–40)	0.016
HGT	Median (IQR)	7.4 (6–11.9)	7.3 (5.7–12.7)	7.9 (6.8–11.8)	0.100
Creatinine	Median (IQR)	74 (58–116)	71 (55–100)	101 (71–262)	< 0.001
CRP	Median (IQR)	145 (76–223)	139 (70–207)	204 (124–285)	< 0.001
White Cell Count	Median (IQR)	8.64 (6.56–11.16)	7.93 (6.35–10.59)	9.42 (7.8–13.32)	0.002
Lymphocytes	Median (IQR)	1.65 (1.22–2.20)	1.68 (1.27–2.22)	1.37 (0.9–1.855)	0.031
Neutrophils	Median (IQR)	5.62 (4.12–8.22)	5.34 (3.97–7.70)	7.3 (5.60–9.35)	0.002
Platelets	Median (IQR)	280 (221–374)	287 (223–374)	273 (207–369)	0.480
HbA1c	Median (IQR)	12.8 (11.2–14)	12.9 (11.3–14.1)	12.0 (10.4–13.4)	0.038

*ARDS* acute respiratory distress syndrome, *ARV* antiretroviral therapy, *CD4* cluster of differentiation 4, CRP: C-reactive protein, IQR: interquartile range, *FiO2* fraction of inspired oxygen, *HGT* Hemo Glucose test, *PaO2* partial pressure of oxygen, *TB* Tuberculosis

^Median (IQR), *CD4 counts: At least six months prior to SARS-CoV-2 infection

### Association of demographic, haematological, and biochemical parameters with mortality among PLWH with SARS-CoV-2 infection

Table 2 shows the association between demographic, haematological, and biochemical parameters with survival among PLWH. Creatinine (1.001, 95% CI: 1.001–1.002, p < 0.001), Neutrophils (1.02, 95% CI: 1.01–1.03; p < 0.001), HbA1c (1.01, 95% CI: 1.007–1.010; p < 0.001), and CRP (1.003, 95% CI: 1.001–1.005, p < 0.001) were significantly associated with the risk of mortality. In multivariable analysis smoking, neutrophils, HbA1c and CRP were all significantly associated with increased risk of mortality (aRR 4.17: 1.50–10.01); (aRR: 1.03, 95% CI: 1.02–1.05; p < 0.001); (aRR: 1.01, 95% CI: 1.001–1.02; p = 0.021); (aRR; 1.002, 95% CI: 1.002–1.01; p < 0.032).

**Table 2:** Univariate and multivariable level analysis of factors associated with COVID-19 mortality among PLWH

Characteristic	RR (95% CI)	p	ARR (95% CI)	p
Age categories				
< 40	1			
40–49	1.96 (0.94–4.07)	0.072	2.24 (0.76–6.64)	0.143
≥ 50	2.88 (1.46–5.67)	0.002	2.32 (0.67–8.14)	0.186
Sex: Female	1.62 (1.04–2.52)	0.032	2.00 (0.85–4.67)	0.111
Smoker	2.86 (1.75–4.69)	< 0.001	4.17 (1.50–11.61)	0.006
Hypertension	1.32 (0.85–2.05)	0.220	0.93 (0.40–2.19)	0.869
Diabetes Mellitus	1.29 (0.82–2.04)	0.271	1.04 (0.51–2.16)	0.904
Chronic Kidney Disease	1.46 (0.73–2.93)	0.283		
TB	1.35 (0.76–2.38)	0.309		
Viral Load: Detectable	1.18 (0.65–2.16)	0.588		
Congestive Heart Failure	1.87 (0.77–4.52)	0.168		
Acute kidney injury	2.11 (1.36–3.30)	0.001	0.71 (0.33–1.57)	0.401
ARDS	1.17 (0.43–3.15)	0.760		
Shock	1.22 (0.37–4.03)	0.742		
Cough	0.88 (0.54–1.45)	0.624		
Fever	0.87 (0.55–1.39)	0.565		
Sore Throat	0.59 (0.23–1.50)	0.265		
Myalgia	0.868 (0.50–1.52)	0.621		
ARV therapy	1.04 (0.46–2.36)	0.922		
*CD4: 200–499	1			
< 200	1.58 (0.94–2.65)	0.085	1.27 (0.62–2.61)	0.509
≥ 500	0.55 (0.22–1.38)	0.202	0.35 (0.09–1.29)	0.114
pa02	1.012 (0.96–1.07)	0.665		
HGT	0.999 (0.973–1.03)	0.944		
CRP	1.003 (1.001–1.005)	< 0.001	1.002 (1.002–1.01)	0.032
Creatinine	1.001 (1.001–1.002)	< 0.001	1.00 (0.99–1.00)	0.187
WCC	1.05 (1.01–1.08)	0.005		
Neutrophils	1.024 (1.01–1.03)	< 0.001	1.03 (1.02–1.06)	< 0.001
HbA1c	1.01 (1.007–1.01)	< 0.001	1.01 (1.001–1.02)	0.011
Platelets	0.999 (0.997–1.00)	0.477		
Lymphocytes	0.882 (0.58–1.34)	0.557		

*ARDS* Acute respiratory distress syndrome, *ARV* antiretroviral, *CD4* cluster of differentiation 4, CRP: C-reactive protein, *HbA1c* Glycated haemoglobin A1, *HGT* haemo glucose test, *PaO2* partial pressure of oxygen, *TB* tuberculosis, *WCC* white cell count

*CD4 counts: At least six months prior to SARS-CoV-2 infection

### ROC curves and cut-offs

**Fig. 2:**
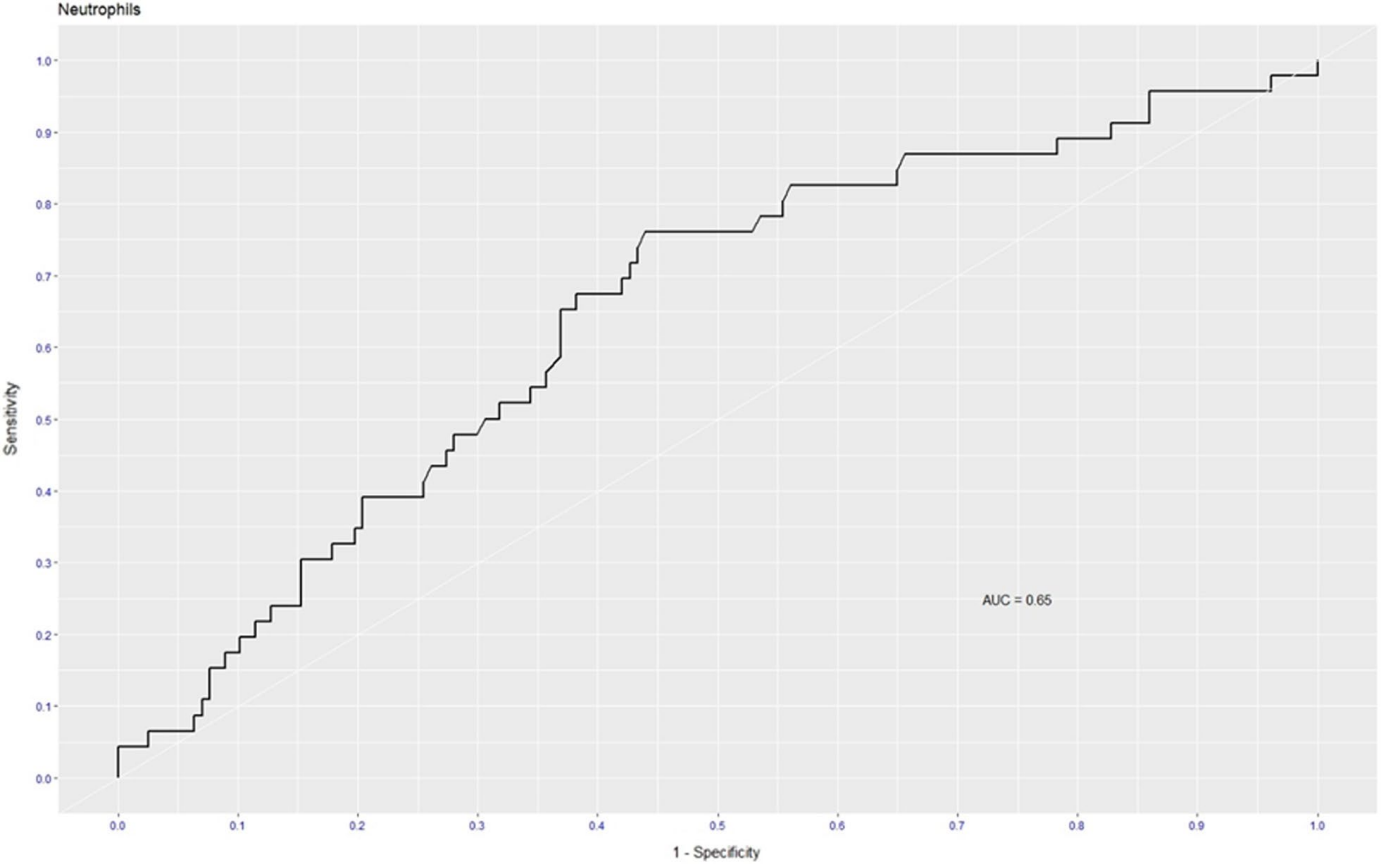
ROC curve for Neutrophils between death and discharged COVID-19 cases with HIV infection

**Fig. 3:**
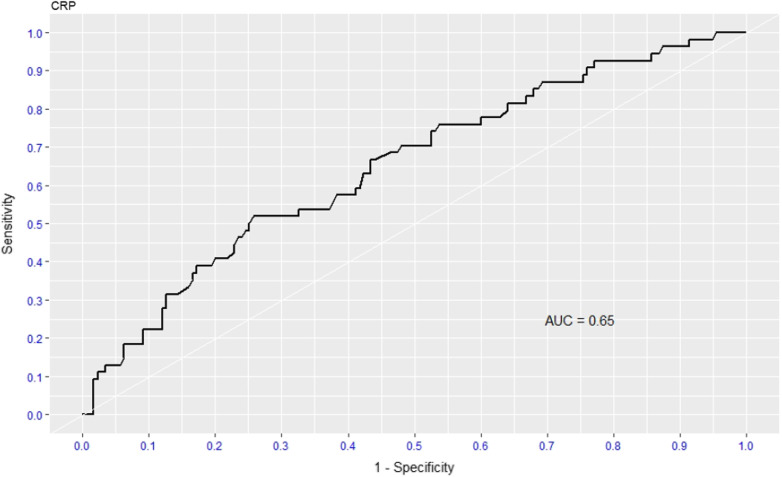
ROC curve for CRP between death and discharged COVID-19 cases with HIV infection

As the adjusted RR was significant for neutrophils and CRP at the borderline, we determined the optimal cut-offs to predict non-survival and test performance of these two parameters using ROC curves. ROC curves were drawn with sensitivity as the horizontal coordinate and the 1‑specificity as the vertical coordinate to predict COVID-19 severity and mortality among PLWH admitted to the hospital. The proposed optimum cut-off points for neutrophils derived from ROC analysis was ≥ 5.6 × 10^9^/L with sensitivity = 76% and specificity = 56% (Table 3 and Fig. 2). An optimal cut-off of 202 mg/L rendered 52% sensitivity and 74% specificity for the CRP (Table 3 and Fig. 3). The area under the ROC curves (AUC) for the neutrophils and CRP were 0.66 and 0.65, respectively (Table 3). However, the performance of both was suboptimal to use as a predictive marker on their own. The combination of the two variables and their predictive value on the roc curve changed slightly to AUC = 0.67.

**Table 3:** Optimal cut-off, sensitivity, specificity, and AUC for CRP, and Neutrophils

Analyte	Direction	Optimal cut point	Sensitivity	Specificity	AUC
CRP (mg/L)	≥	202	0.52	0.74	0.65
Neutrophils (× 10^9^/L)	≥	5.6	0.76	0.56	0.66

### Kaplan–Meier survival estimates between males and females

**Fig. 4:**
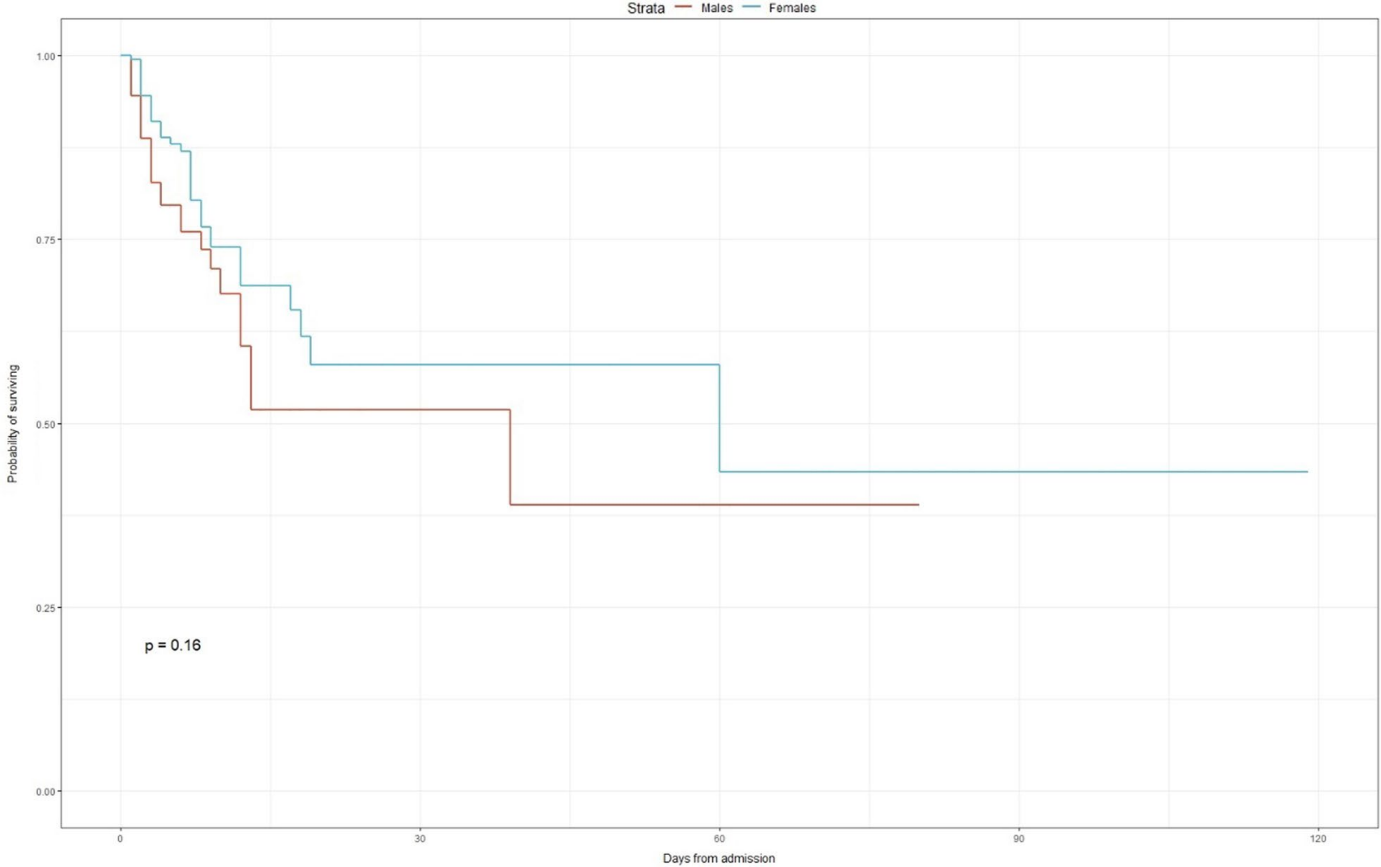
Kaplan–Meier plot for overall survival among COVID-19 males and females with HIV co-infection

**Fig. 5:**
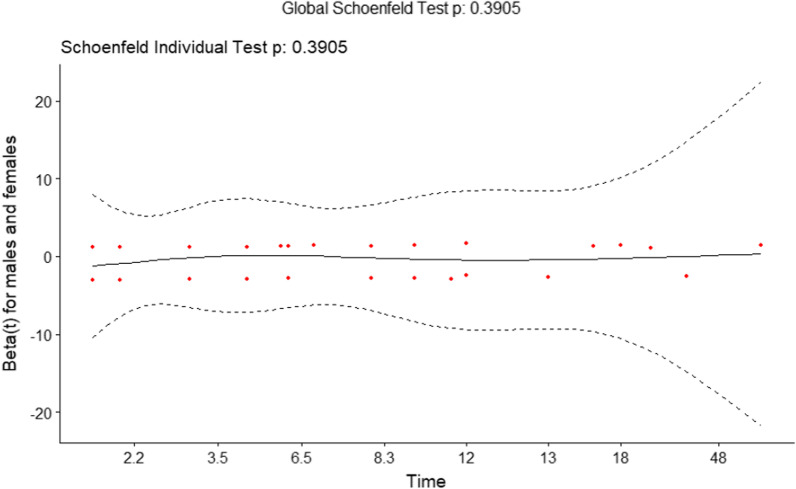
Plots of the scaled Schoenfeld residuals of each covariate between males and females

The rate of death seemed to be higher among male patients during the whole duration of the hospitalization. However, Poisson regression analysis was used to compare male and female mortality, no significant difference was found (p = 0.160) (Fig. 4). The median stay was 7 (IQR: 3–12) days for females compared to 5 (IQR: 3–9.5) days for males. The overall median stay was 6 (IQR: 3–10) days. (Fig. 4). The plots of the scaled Schoenfeld residuals of each covariate against log-time were used to determine whether the proportional hazards assumption was violated (Fig. 5). The Schoenfeld residuals test revealed that a proportional hazard assumption had the same effect on male and female survival rates (p = 0.3905) (Fig. 5).

## Discussion

This retrospective cohort study of 255 PLWH with SARS-CoV-2 infection focused on hospitalized individuals at a district hospital in Cape Town. Most of these patients were stable on ART and over one-third of them died. The median (IQR) age was 46 (37–54) years, with 70.9% of the population being female. PLWH who died were older than 50 years old, females, smokers, had acute kidney injury, CD4 less than 200 mm3, hypoxemic, high CRP, and HbA1c. Creatinine, Neutrophils, HbA1c, and CRP were significantly associated with the risk of mortality in univariable analysis. Smoking, neutrophils, HbA1c, and CRP were all significantly associated with an increased risk of mortality in a multivariable analysis. CRP and neutrophil performance were both suboptimal for use as survival predictors. The high mortality experienced among PLWH was consistent with a systematic review and meta-analysis of twenty-two studies involving 20,982,498 PLWH, which found that HIV was associated with a significantly higher risk of SARS-CoV-2 infection (RR 1.24, 95 percent CI 1.05–1.46) [6]. The overall pooled RR of COVID-19 mortality associated with HIV was 1.78 (95%CI 1.21–2.60), implying that HIV-positive patients have an 80% increased risk of death when compared to people who do not have HIV/AIDS [6]. Similarly, a review revealed that the RR of severe COVID-19 in PLWH was significant only in Africa (RR = 1.14, 95% CI = 1.05–1.24), while the relative risk of mortality was 1.5 (95% CI = 1.45–2.03) globally [33]. Although the RR of severe COVID-19 in PLWH appeared to be lower, the studies included in this review were conducted in often well-resourced and skilled tertiary, academic, and intensive care unit (ICU) hospital settings. The high COVID-19 mortality rate among PLWH should be considered in the context of the devastation brought on by the COVID-19 pandemic in a limited resource district hospital which is already overwhelmed by the high burden of HIV, TB, and other non-communicable diseases. In addition to this, the low CD4 count, high AIDS rate, and a high prevalence of multiple co-morbidities among PLWH with COVID-19, seem to have a key role in the high COVID-19 mortality in a district hospital setting. A recent study conducted among PLWH in Cape Town revealed that PLWH with CD4 count < 200 cells/µl were associated with COVID-19 death (aHR vs people living without HIV, 2.36 [95% CI, 1.47–3.78]; aHR vs PLWH with CD4 count ≥ 350 cells/µl, 1.97 [95% CI, 1.14–3.40]) [34]. Compared with HIV disease stage 1 (CD4 counts > 500 cells/ml), hospitalization rates were 29% higher for stages 2 (CD4 counts 200–499 cells/ml) and 69% higher for stage 4 CD4 counts < 200 cells/ml) [35]. The unadjusted hazard ratio of COVID-19 death was 1.07 (95% CI 0.88–1.32) for HIV-positive vs. HIV-negative, and 2.14 (95% CI 1.70–2.20) after adjustment for age, sex, and other comorbidities [34]. This finding was consistent with our findings, as CD4 counts of less than 200 cells/ml were associated with COVID-19 among PLWH in unadjusted RR. The interplay between the inverse relationship between the CD4 count, HIV viral load, the access and adherence to ARV therapy play a crucial role in the determinant of severity of HIV infection and associated outcomes.

As this study was conducted in a high HIV/TB setting, emphasis is also placed on the interactions between COVID-19, HIV, and TB. This cohort found 12.9% cases of TB coinfection with COVID-19 among PLWH. However, the records did not specify whether these cases were active or had previous TB. Knowing that a study performed in an urban township of Cape Town observed a very high (88.0%) latent TB infection (LTBI) prevalence rate among PLWH [36] and SARS-CoV-2 infection decreases TB–specific CD4 + T cell response [8], many concerns have been raised about the possibility that COVID-19 could reactivate latent TB in a high TB-endemic setting such as Khayelitsha district. The reactivation of TB could be explained by cytokines and chemokines dysregulation, higher consumption of CD4 + and CD8 + T-cells, decrease in regulatory T-cells, and an altered innate immune environment leading to a cytokine storm and worsen tissue damage [37–39]. A study showed poor outcome in mortality rate among COVID-19/HIV/TB co-infection compared to COVID-19/TB [7]. Even though, our study found no significant link between COVID-19 mortality and HIV/TB cases, a large cohort study conducted in Cape Town found an association between COVID-19 mortality and previous and active TB [34]. Our findings should be interpreted in the context of inadequate TB screening in COVID-19 patients admitted to the district hospital.

Our study also found out that raised neutrophil count, CRP, HbA1c, and smoking were associated with worse outcomes after confounding adjustment. This is substantial as those biomarkers may play a vital role in preventing worse outcomes among COVID-19 in PLWH. A multivariable analysis showed that raised neutrophil count was an independent risk factor for mortality. This finding is similar to the previous study which revealed that a higher neutrophil count in COVID-19 patients was found to be associated with a higher mortality rate among PLWH with SARS-CoV-2 infection [40]. This could be explained by emergency release of granulocyte colony-stimulating factor with activation of immature neutrophils, neutrophil maturation, neutrophil degranulation, and release of neutrophil extracellular traps (NETs) [41, 42]. Neutrophil activation and degranulation result in release of neutrophil-activation markers including resistin, lipocalin-2, hepatocyte growth factor and interleukin-8 which causes significant collateral damage. The immune response of natural killer cells and T lymphocytes contributes to the formation of NETs and the activation of the complement system (C5 and C3) [42]. The result is the development of microvascular thrombosis, which leads to organ damage with an increased risk of severe COVID-19 and mortality [41, 42]. The early elevation of activated immature neutrophils, G-CSF and neutrophil-activation markers were suggested as early predictors of severe COVID-19 infection and increased mortality. Additionally, alteration to gene expression of neutrophils with a prominence of immature neutrophils markers was found in severe COVID-19 patients [43]. While access to these tests is not feasible in limited-resource countries the use of neutrophil count could be used as an affordable predictor variable of COVID-19 severity and mortality among PLWH. Importantly, our analysis revealed a significantly higher CRP in the non-survivor group compared to the survivor group. The CRP is an acute phase reactant which functions as a reliable biomarker and is recommended as a predictor of COVID-19 severity and mortality. Our findings were in the line with studies including PLWH and SARS-COV-2, conducted in the same settings which showed significantly high CRP levels among non-survivors [8, 40]. The cut-offHbA1c level of ≥ 6.5% is defined as diabetes mellitus. A study revealed that high HbA1c level is associated with inflammation, hypercoagulability, and low SaO2 in COVID-19 patients, and the mortality rate is higher in patients with diabetes mellitus [44, 45]. In our study, HbA1c was high in both discharged and death cases and the difference in HGT was not statistically significant between the two groups. The plausible explanation for the high HbA1c among COVID-19 cases with HIV could be more severe inflammatory process and more intense coagulation disorder associated with poor prognosis [45]. Another explanation could be the high HbA1c found in public sector primary health care facilities in Cape Town as a study showed 78.9% of type 2 diabetes mellitus patients had HbA1c outside of the target range [46]. As high HbA1c may not be a reliable biomarker in this context, our findings were focused on the neutrophils and CRP. Even though, the performance of both was suboptimal to use as a prediction marker, high neutrophils and CRP are consistent with previous studies showing higher diagnostic accuracy and AUC of ROC analysis [47, 48]. Lastly, our study found that smoking was an independent factor of mortality PLWH with SARS-CoV-2 infection. A systematic review of fifty‐seven studies found that current smokers are at reduced risk of SARS-CoV-2 infection, while former smokers appeared to be at increased risk of hospitalization, increased disease severity and mortality from COVID-19 [49]. We could not find a plausible explanation for this association. However, a study conducted on smoking status found that the response rate was low among current smoking (10%) compared to 58.8% among former smokers [50]. This could overestimate the effect of smoking status on COVID-19 outcome. Even though our study did not identify current and former smoking status, the association between smoking and COVID-19 mortality among PLWH should be taken with caution.

This study has several strengths. This is the first study to our knowledge conducted in a South African district-level hospital involving COVID-19 patients with HIV infection. Based on the study findings, COVID-19 management among PLWH at the district-level may face multiple challenges due to limited resources. As primary healthcare systems are often poorly funded, overcrowded, short-staffed, and are unable to provide efficient care. This study has provided an overview of COVID-19 severity and mortality risk factors among vulnerable and specific population of PLWH in district-level hospital. Additionally, significant COVID-19 challenges faced by the facility included the lack of infrastructure within the facility with a daily bed occupancy rate to deal with the separation of COVID-19 patients from Persons Under Investigation (PUIs) and general medical admissions. This study showed that easily available biomarkers in a district hospital setting are associated with COVID-19 mortality. These biomarkers could be used by clinicians at district-level hospitals to risk stratify and motivate for higher level of care at their tertiary referral hospitals. This study had several limitations among which is its retrospective design nature. The retrospective cohort study design limited our ability to gather data about factors that may influence the risk of mortality such as the HIV viral load, ART regimens, active and previous TB, estimated glomerular filtration rate (eGFR), and smoking status. Another study flaw was that transferred cases were not followed up with the Western Cape Provincial Health Data Centre (PHDC) to learn about their outcomes. This was the source of missing and incomplete data. The other limitation of the study might be that it is a single centre study but also underpowered due to sample size used, hence our results might be limited to the population of Khayelitsha and not generalized to other similar populations.

## Conclusion

This study showed that the mortality rate of COVID-19 patients co-infected with HIV was high at district hospital level. An increase in neutrophils, HbA1c, and CRP were all significantly associated with risk of mortality. As such COVID-19 diagnosis and management among PLWH at the district-level should include easily accessible, reliable, and affordable assessment for these biomarkers to support the front-line clinicians for an effective risk stratification of patients at an increased of poor outcome and subsequent early transfer to tertiary level hospital for advanced care and minimise loss of life in such patients.

## Data Availability

All data are available upon request from the corresponding author of the study.

## Abbreviations

AIDS: Acquired immunodeficiency syndrome
ARDS: Acute respiratory distress syndrome
ART: Antiretroviral therapy
ARV: Antiretroviral
AUC: Area under the curve
BMI: Body mass index
CDC: Centers for diseases control and prevention
CD4 count: Cluster of differentiation 4
CO2: Partial pressure of carbon dioxide
COVID-19: Coronavirus disease 2019
Cr: Serum creatinine
eGFR: Estimated glomerular filtration rate
ELISA: Enzyme-linked immunoassay
FiO2: Fraction of inspired oxygen
ICU: CRP: C-reactive protein
HbA1c: Glycated haemoglobin
HGT: Hemo Glucose test
HIV: Human immunodeficiency virus
ICU: Intensive care unit
LTBI: Latent TB infection
NET: Neutrophil extracellular traps
N/L Ratio: Neutrophil to lymphocyte ratio
PCT: Procalcitonin
P/F Ratio: Fraction of inspired oxygen ratio
PCR: Polymerase chain reaction
pH: Potential hydrogen
PLWH: People living with HIV
PUIs: Persons Under Investigation
ROC: Operating characteristic curve
SA: South Africa
SANAS: South African National Accreditation Services
SARS-CoV-2: Severe acute respiratory syndrome coronavirus 2
TB: Tuberculosis
WCC: White cell count
WHO: World Health Organization
